# Assessing the impact of Arctic shipping routes on the global container shipping network’s connectivity

**DOI:** 10.1080/1088937X.2024.2399775

**Published:** 2024-09-11

**Authors:** Mark Ching-Pong Poo, Zaili Yang, Yui-yip Lau, Pisit Jarumaneeroj

**Keywords:** Maritime transportation, Arctic shipping, network analysis, global container shipping network

## Abstract

Amidst the intensifying impact of climate change, the extension of navigable periods along Arctic Shipping Routes (ASRs) has garnered attention as a maritime route for container vessels. The urgency to comprehend the reverberations of ASRs on the global container shipping network (GCSN) led to the development of the Latitudinal Centrality Index (LCI), which integrates latitude and centrality in maritime analysis. This index evaluates ASRs’ influence across 968 port nodes within the GCSN. By exploring scenarios encompassing seasonal fluctuations over the years, this study delves into the sway of ASRs compared to a benchmark state devoid of ASR engagement. The study’s revelations highlight a discovery: the assimilation of ASRs augments interconnectivity, or resilience, within the GCSN. The GCSN thrives as a cohesive and adaptable entity upon full integration of ASRs, indicating a promising trajectory for global container shipping.

## Introduction

Due to the impacts of global climate change, specific Arctic regions have experienced an extended duration of ice-free conditions in the ocean. These areas have seen warming rates approximately twice as fast as the global average, as documented by Boylan (2021). Consequently, this has resulted in thinner sea ice, later onset of freezing in autumn, and an earlier onset of melting. Remarkably, these alterations have contributed to the expansion of Arctic shipping routes, as emphasized by Cao et al. (2022). Yet, Panahi et al. (2021) and Chen et al. (2021) addressed that sea ice melting was an enabler rather than a trigger to the expansion of Arctic shipping. Since 1979, Arctic sea ice has consistently reached its minimum extent in September, diminishing steadily each year. A striking example of this trend occurred in 2012 when Arctic sea ice reached an unprecedented low of 3.4 × 10^6^ square kilometers. This decline was primarily attributed to intense storms in the central Arctic, as indicated by Liu et al. (2021).

According to projections from the Intergovernmental Panel on Climate Change (IPCC), ice coverage in Northern Hemisphere seas is expected to further diminish to 1.5 × 10^6^ square kilometers by 2025, as Stocker et al. (2013) outlined. Moreover, by 2050, it is anticipated that the Arctic Ocean will be ice-free for approximately six months during the summer. In such a scenario, the Northwest Passage (NWP) is expected to be ice-free on an annual basis for a period of two to four months, while the Northern Sea Route (NSR) is projected to be accessible for three to six months each year, as reported by Stewart et al. (2020) and Wagner et al. (2020).

As has been illustrated, melting Arctic sea ice has significantly increased the likelihood of commercializing these so-called Arctic shipping routes (Guo et al., 2022; Liu et al., 2021), with increased shipping activities (Prentice et al., 2021; Theocharis et al., 2018). To this end, between 2013 and 2019, there was a 75% rise in the distance traveled by various ships and a 25% rise in the number of vessels involved in Arctic shipping (Boylan, 2021).

In terms of trading, the commercialization of Arctic Shipping Routes (ASRs) can drastically redefine the geographical dynamics of global maritime transport networks, as they introduce new maritime connections while modifying the existing trade routes in various regions – including those in the Baltic, the Arctic, Northwest Europe, and Northeast Asia – at the same time. For instance, the transit distance from Northeast Asia to Northwestern Europe could be reduced by 4,900 nautical miles through the NWP, compared to the traditional trade routes through the Panama Canal or the Suez Canal. Likewise, the transit distance from Northeast Asia to Northeastern America could be reduced by 2,500 nautical miles through the NSR (Cao et al., 2022). Nevertheless, Arctic transit traffic remains minimal, even today. Destination shipping is expanding, but transit does not follow up for reasons as underlined in the literature: seasonality, lack of just-in-time possibility, risks, and difficulty in developing a business model that will give shipping companies confidence in the profitability of ASR. These natures also have limits rooted in business strategies nurtured by shipping companies (Gunnarsson, 2021; Lau et al., 2023; Panahi et al., 2021). Furthermore, ASR is not an issue of technology: ice-classed vessels can be ordered, and Baltic-ice-classed vessels are likely to be able to play Arctic waters soon in the summer; higher ice-classed vessels can already do it year-round but cost much more to build and operate.

With potential benefits, Arctic shipping has garnered considerable attention from researchers, decision-makers, policymakers, international organizations, industrial practitioners, environmentalists, and logistics associations in the past decade (Theocharis et al., 2018). The interest in exploring the challenges and benefits of ice-free seasons has also grown among corporations and governments (Panahi et al., 2021), which, in turn, sheds light on the investigation of potential ASR impact on the global container shipping network (GCSN) in recent years.

Among the existing research, network analysis is the most widely applied instrument for studies related to maritime transport networks, as it helps provide a better understanding of the network and port levels concurrently. Nonetheless, most previous studies have primarily focused on developing centrality measures in port systems. While it is indisputable that these multiple centrality measures help understand port systems and their respective roles within the networks, they are, however, improper for the case of ASR analysis due largely to the unique characteristics and evolving dynamics of ASRs (Ducruet & Notteboom, 2012; Lam & Yap, 2011; Lau et al., 2023; Panahi et al., 2021). In light of this gap, there is a need to investigate the changing structure of GCSN and the specific positions of ports therein due to the ASRs. For this purpose, a novel Latitudinal Centrality Index (LCI), which takes into account geographical locations and network locations of ports in the GCSN, is devised to evaluate the impact of ASRs on the GCSN.

It should be remarked that, as the participation of international transport actors has significantly altered the spatial characteristics of maritime networks (Ducruet et al., 2020), the choices of centrality measure, including the LCI, should be determined by specific research objectives, parts of the port system under study, and research questions being addressed. A combination of centralities is among viable choices that enable researchers to capture different dimensions of centrality and gain more profound insights into the network structure and its internal dynamics (Poo & Yang, 2022; Wan et al., 2021) – although the GCSN is relatively stable compared to other transportation networks like roads and railways, as Ducruet et al. (2018) and Peng et al. (2018) noted.

Our study aims to assess the network’s importance and structure using multiple centrality measures: degree centrality, closeness centrality, and betweenness centrality. Our analysis will also incorporate the LCI. This approach allows us to address a gap in the existing research and simultaneously gain valuable insights into the dynamics of the GCSN and its organization. By combining the LCI with a multi-centrality assessment, we can better understand how global container shipping routes are structured and identify critical locations in facilitating this transportation network. We expect that the findings of this study would be of paramount importance to the current knowledge of shipping operations – especially in the Arctic region – which, in turn, creates a future research agenda, along with the exploration of potentially new markets along the ASRs. The results of this study will also contribute to a more comprehensive understanding of the role of Arctic shipping in the GCSN, enabling informed decision-making for stakeholders involved in Arctic shipping operations and infrastructure development.

The paper’s organization can be broken down as follows: In Section 2, we conduct an extensive review of the existing literature, where we delve into the latest advancements and insights regarding the potential utilization of ASRs and the development of various centrality metrics. Moving on to Section 3, we delve into the intricate details of our research methodology. This section provides a comprehensive account of our study’s approach, including the creation of a GCSN featuring 968 ports, the calculation of the LCI, a demonstration of its transformative impact on the established paradigm, and an evaluation of the network’s behavior before and after the integration of ASRs. Sections 4 and 5 are dedicated to presenting our primary discoveries and drawing conclusions from the research. These sections encapsulate in-depth discussions and the implications derived from our computational findings.

## Literature review

The literature review section is split into three sub-sections. The first subsection mentions the development of ASRs, and the second subsection describes the development of studies on port centrality.

### Development of Arctic shipping routes

**Figure 1.**
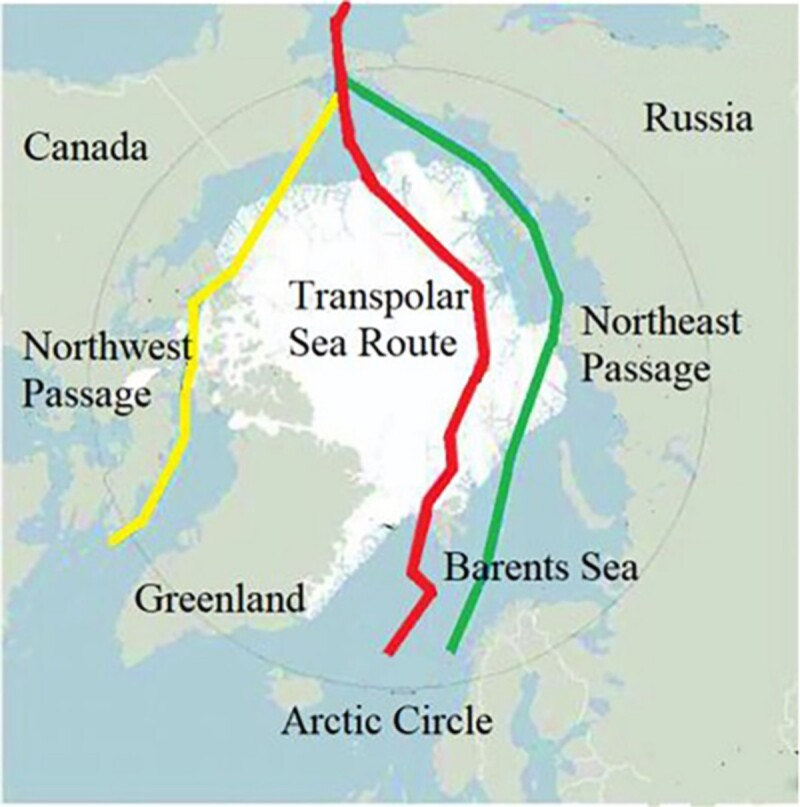
Overview of Arctic shipping routes (Lin et al., 2024).

There is much debate among the media and the scientific community regarding developing Arctic Shipping Routes (ASRs). The maritime industry has been somewhat reluctant to develop these routes, leading Russia to attempt to set up a new business model with transhipment hubs to attract cargo despite the low interest among shipping companies for transit (Lasserre & Cyr, 2022). These endeavors have paved the way for the evolution of ASRs, pictorially captured in Figure 1.

Figure 1 provides an overview of ASRs, which typically comprise three main shipping lanes: the Northern Sea Route (NSR), the Northwest Passage (NWP), and the Transpolar Sea Route (TSR). The NWP is the sea lane between the Pacific and Atlantic oceans via the Arctic Ocean, along the northern coast of North America via waterways through the Arctic Archipelago of Canada. Conversely, the NSR, governed by Russia, is the shortest shipping route between the Asia-Pacific region and the western part of Eurasia, supported by continuous investment in icebreakers and natural resources terminals like Sabetta, Arctic Gate, and the future terminals near Dickson (Melia et al., 2016).

Compared to the NSR, the NWP accounts for only a small portion of international trade due to its lack of predictability (Fu et al., 2021). Utilizing the NWP requires additional maritime infrastructure and deep-water ports capable of accommodating large vessels, contrasting the predominantly rural villages along its route. The surge in Arctic traffic volume can be primarily attributed to coal transportation, liquefied natural gas, crude oil, and ongoing exploration of natural resources in the Russian Arctic. The cargo volume of the NSR has significantly grown from 10.7 million tons in 2017–31.5 million tons in 2022 (Erokhin et al., 2022; Li et al., 2021).

The Transpolar Sea Route (TSR), often referred to as the ‘Silk Road on Ice’ (Guo et al., 2022), has garnered significant global and domestic attention. Although still a hypothetical route, it is considered a viable alternative through the Arctic region, with comprehensive development expected after the NWP and the NSR (Boylan, 2021). Due to the reduction and diminishing thickness of Arctic sea ice, it is anticipated that ships without ice-class capabilities can traverse the NSR and NWP before 2050 (Chen et al., 2021; Lau et al., 2023; Prentice et al., 2021). By the end of the twenty-first century, the NWP may be without ice cover for two to four months, while the NSR could remain ice-free for three to six months.

Despite the potential, the development of ASRs faces several challenges. The profitability of ASRs is often questioned due to unpredictability associated with political, economic, and technical instabilities and various hazards (Afenyo et al., 2017). However, some research indicates benefits such as enhanced accessibility, reduced transit times, improved network connectivity, lower greenhouse gas emissions, and reduced operational costs (Chen et al., 2021; Lasserre et al., 2016; Theocharis et al., 2018). There is also a need to examine the effects of pollution in the Arctic region, with studies forecasting emissions trends and possible sea ice conditions in various scenarios (Peters et al., 2011; Winther et al., 2014).

Feasibility studies comparing Arctic shipping with standard routes, such as those passing through the Panama Canal and the Suez Canal, highlight the challenges and risks associated with ASRs (Cariou et al., 2021; Wan et al., 2018). Furthermore, diversifying commodities transported through these routes could support novel business models and emerging maritime routing choices (Munim et al., 2022). However, the existing facilities fail to meet fundamental navigation, rescue, and cargo handling requirements, especially for larger vessels (Celik & Van Hassel, 2023; Jiang et al., 2021).

While Arctic shipping routes promise future global connectivity, their commercial feasibility remains uncertain and requires further advancements and strategic planning. Governments, policymakers, maritime logistics firms, and Arctic stakeholders must consider the latest ASR shipping activities and their implications for global connectivity. The primary objective of this work is to evaluate the feasibility of ASRs if they become accessible during certain parts of the year. This includes analysing these routes’ economic, environmental, and technical aspects and their impact on global shipping networks and the Arctic region’s ecological stability. By focusing on these areas, this research aims to provide a comprehensive multi-centrality assessment of the viability and sustainability of ASRs, offering valuable insights for stakeholders and policymakers involved in Arctic maritime activities.

### Research on port centrality

Ports are vital in global trade and transportation, serving as virtual nodes within complex maritime networks (Panahi et al., 2022). Understanding the centrality of ports within these networks is crucial for analysing their connectivity, accessibility, and overall importance. Centrality measures could also provide valuable insights into port systems and their internal dynamics, enabling informed decision-making in port planning, logistics optimization, and network analysis. In this context, several research studies have explored various centrality measures to assess and compare the criticality of ports within maritime networks, as summarized in Table 1.

**Table 1.** Research studies on the centrality of ports.

Journal reference	Degree	Closeness	Betweenness	Other
Ducruet (2013)	˅		˅	
Du et al. (2014)	˅			
Tovar et al. (2015)	˅	˅	˅	
Fraser et al. (2016)			˅	
Wang and Cullinane (2016)	˅	˅	˅	
Bartholdi et al. (2016)				Eigenvector centrality
Fugazza and Hoffmann (2017)	˅			
Ducruet and Wang (2018)	˅			
Jeon et al. (2019)	˅		˅	
Wu et al. (2019)	˅	˅	˅	
Cheung et al. (2020)				Eigenvector centrality
Wan et al. (2021)	˅	˅	˅	
Zhang et al. (2022)			˅	
Wen et al. (2022)				Neighbourhood-based centrality, Iterative refinement centrality
Dirzka and Acciaro (2022)	˅		˅	
Wang et al. (2022)	˅			
Liu et al. (2022)		˅		
Jarumaneeroj et al. (2023) and Jarumaneeroj et al. (2024)				Eigenvector centrality
Wan et al. (2022)	˅	˅	˅	

Degree centrality is a metric that quantifies a port’s connections with other ports in a network, indicating its level of direct linkage. Several studies in Table 1 have focused on examining degree centrality, underscoring its importance in comprehending the connectivity and significance of ports within a network. Notable studies that investigate degree centrality include Ducruet (2013), Du et al. (2014), Tovar et al. (2015), Wang and Cullinane (2016), Fugazza and Hoffmann (2017), Jeon et al. (2019), Wu et al. (2019), Wan et al. (2021), Dirzka and Acciaro (2022), Wang et al. (2022), and Wan et al. (2022).

Closeness centrality, on the other hand, evaluates a port’s proximity to all other ports in terms of the shortest path length, reflecting its accessibility and efficiency in reaching other ports within the network. Multiple studies have recognized the significance of closeness centrality as a metric for analysing port centrality. These studies include Tovar et al. (2015), Wang and Cullinane (2016), Wu et al. (2019), Wan et al. (2021), Liu et al. (2022), and Wan et al. (2022).

Another extensively examined measure is betweenness centrality, which measures the extent to which a port acts as a bridge or intermediary in the flow of goods or information between other ports. It identifies ports that are critical in connecting different parts of the network. Several studies have explored betweenness centrality to gain insights into the pivotal position of ports within a network. Noteworthy studies that investigate betweenness centrality include Ducruet (2013), Tovar et al. (2015), Fraser et al. (2016), Wang and Cullinane (2016), Jeon et al. (2019), Wu et al. (2019), Wan et al. (2021), Zhang et al. (2022), Dirzka and Acciaro (2022), and Wan et al. (2022).

While degree centrality, closeness centrality, and betweenness centrality are the most commonly studied centrality measures in port research, a few studies have explored other measures, such as eigenvector centrality, neighbourhood-based centrality, and iterative refinement centrality.

Bartholdi et al. (2016) introduced a variation of eigenvector centrality known as the Container Port Connectivity Index (CPCI) to measure the significance of ports in inbound and outbound trade flows. Building on this, Jarumaneeroj et al. (2023) and Jarumaneeroj et al. (2024) delved deeper into the CPCI, breaking it down into five key metrics, including the number of companies, number of ships, number of services, the largest capacity of ships and cumulative ship capacity in TEUs. This nuanced approach enabled the researchers to offer more comprehensive insights, particularly concerning major economic events in the GCSN, like the Panama Canal’s enlargement and the downfall of Hanjin shipping. Similarly, Cheung et al. (2020) studied port centrality, drawing from eigenvector centrality principles. They determined a port’s centrality based on its connections with other pivotal ports. While using eigenvector centrality is not common in the referenced studies, its application underscores the variety of methods available for port system analysis. In addition to eigenvector centrality, Wen et al. (2022) explored the application of neighbourhood-based and iterative refinement centrality measures to evaluate the susceptibility of the Asia-Europe maritime transportation network. In their setting, neighbourhood-based centrality captured the influence of a port’s direct neighbors in determining its centrality, while iterative refinement centrality iteratively refined centrality scores based on the centrality of neighboring ports. The author found that these centrality measures have proven themselves helpful in offering alternative perspectives of port centrality in the underlying network.

It is important to note that the limited appearance of these less commonly used centrality measures in the literature does not imply their inferiority or lack of importance. Instead, it underscores diverse approaches and methodologies researchers employ to comprehend the dynamics of port systems. By exploring a wide range of centrality measures, researchers can better comprehend port centrality, encompassing connectivity, accessibility, importance, and flow dynamics.

### Research gaps

This study addresses two critical research gaps in Arctic Shipping ASRs and GCSN. Firstly, the research clarifies the addressed knowledge gaps, particularly in the context of the feasibility and sustainability of ASRs. The study explicitly outlines issues related to the validation of port centrality metrics and the detailed examination of economic, environmental, and technical aspects of ASRs. This clarity provides a well-defined foundation for future studies, ensuring a targeted approach to advancing the field.

Secondly, the methodology incorporates rigorous testing and validation of centrality measures such as degree, closeness, and betweenness centrality within evolving global maritime networks. By comparing traditional shipping routes with new ASRs, the research provides empirical evidence to ensure these metrics accurately reflect the dynamics and connectivity of modern shipping networks. This approach helps to substantiate the applicability and reliability of centrality measures in the context of ASRs.

## Methodology

**Figure 2.**
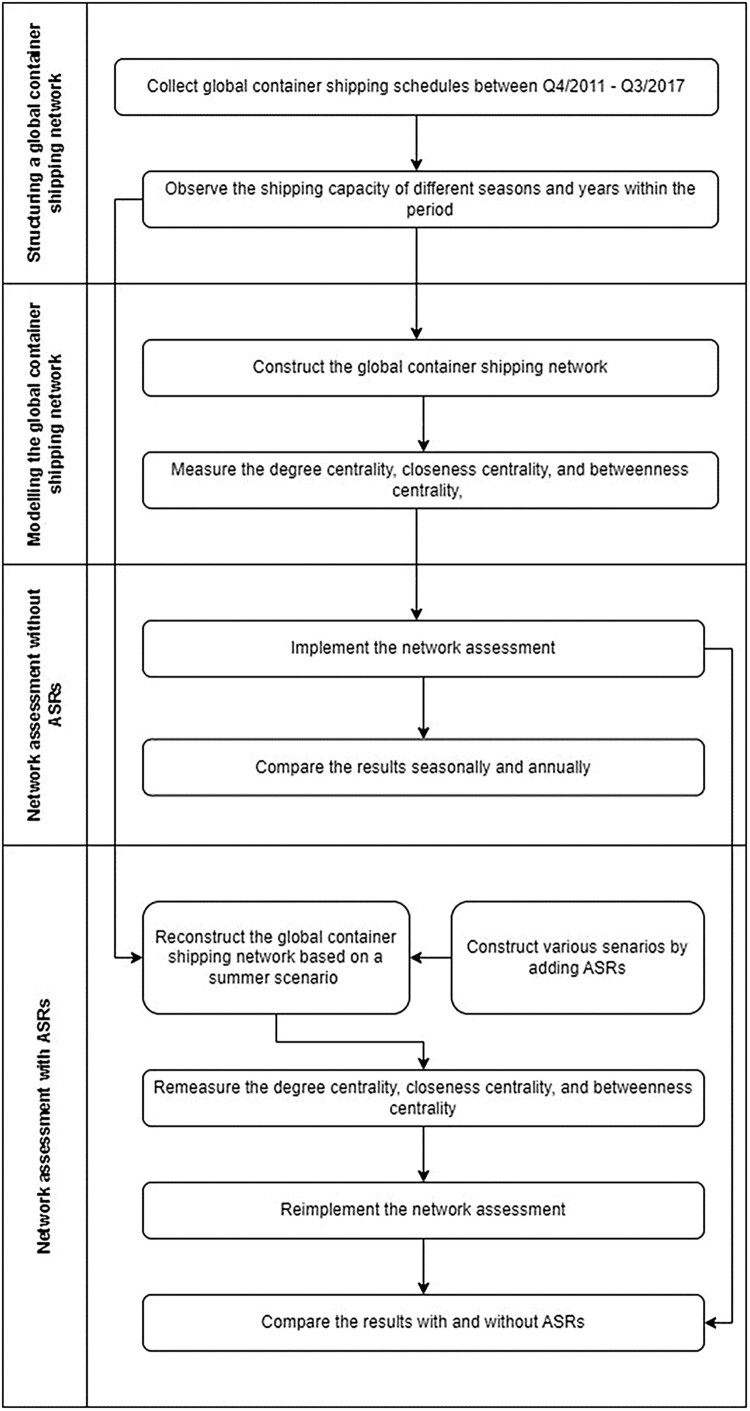
A flow illustrating the proposed methodology.

The methodology for the GCSN assessment in this study is developed by using the classical port centrality analysis (e.g. Poo & Yang, 2022) as the foundation from which new LCI of the ports potentially actively engaged in ASRs are incorporated to evaluate the impact of ASR on GCSN. More specifically, the GCSN without ASRs is first constructed as a baseline for analysing different ASRs, which has implications for the modified GCSN with ASRs. The analysis of these two different networks’ network resilience and port centrality will then be conducted and compared under various scenario settings. For ease of understanding, Figure 2 illustrates the flow of our proposed methodology.

The flow begins by gathering global container shipping schedules from Q4/2011 to Q3/2017. This data is then used to analyse shipping capacity across different seasons and years during this period. Next, the global container shipping network is created, and network metrics such as degree centrality, closeness centrality, and betweenness centrality are calculated.

The methodology is divided into two main parts: network assessment without ASRs and network assessment with ASRs. In the first part, the network is assessed without including ASRs, and the results are compared seasonally and annually. The second part involves reconstructing the shipping network using a summer scenario and incorporating various scenarios with ASRs. The centrality metrics are recalculated, and the network is reassessed. Finally, the results with and without ASRs are compared to determine the impact of ASRs on the global container shipping network. This structured approach thoroughly evaluates the network's dynamics under different conditions.

### Constructing a global container shipping network

BlueWater Reporting (https://www.bluewaterreporting.com/) collects the data for this study, focusing on the global movement of scheduled container vessels. The collection period spans from the fourth quarter of 2011 to the third quarter of 2017, resulting in 55,824 movement data from 24 quarterly data sets.

**Figure 3.**
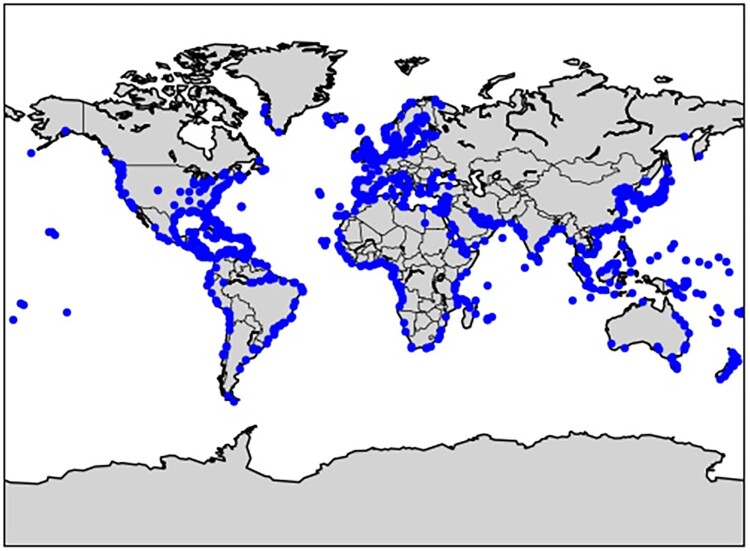
Locations of 968 container ports in this study.

It is important to note that the analysis does not incorporate future data, primarily because of the absence of recent years’ data and the influence of recent economic events, such as the COVID-19 pandemic. Nevertheless, it is feasible to integrate such data into this framework in the future without requiring substantial changes to the methodology. All possible origin-destination (OD) pairs are generated according to the data set, where 968 container ports are identified as either origins or destinations. The locations of these 968 container ports are illustrated in Figure 3. In addition to port nodes, each OD pair’s average weekly TEU (Twenty-foot Equivalent Unit) capacity is also attached to the GCSN, representing the shipping route’s link weight.

Since the data are collected quarterly, it is possible for us to comprehend the variations and differences of GCSNs across different periods – each of which is herein referred to as the seasonal network, for ease of discussion. The data within these seasonal networks corresponds to each of the four seasons in a year: winter (December – February), spring (March – May), summer (June – August), and autumn (September – November). Based on this setting, five annual networks could be constructed for the years 2012, 2013, 2014, 2015, and 2016. For further port details, please refer to Appendix 1, which contains comprehensive information regarding the name, country, subregion, region, latitude, and longitude of ports in this study.

### Modeling the global container shipping network

Once the GCSNs are constructed, they are then analysed by UCINET 6 – a widely adopted program for visualizing and analysing large and complex networks. The justification of UCINET 6 in this study is based on its successes in previous research, such as Poo and Yang (2022). With UCINET 6, we can visualize and analyse the GCSNs, while gaining insights into their properties and characteristics – including the three traditional centrality measures mentioned earlier. The algorithms of calculating three centrality measures are presented in Appendix 2, with more information documented in Poo and Yang (2022).

### Network assessment without ASRs

A common multi-centrality approach in assessing the network (please refer to Poo and Yang (2022) and Wan et al. (2021) for further information) is first used to develop and anlayse the baseline GCSNs in this study. Based on this baseline GCSN, the new indicator, namely the LCI, is developed to capture the central location of the GCSN during different periods.

The computation of LCI is based on a multi-centrality scoring scheme in which the overall rank score ($S_{i}$) of a port – *i.e.* the significance of a port to the whole shipping network – is first computed by Eq. (1) – (4), where $R_{D}(i),\;\;\;R_{C}(i),\;\;R_{B}(i)$ denote the rank of port *i* by degree centrality, closeness centrality, and betweenness centrality in the whole ports involved in the GCSN respectively.

(1) $$S_{D}(i)=P+1-R_{D}(i),\;$$

(2) $$S_{C}(i)=P+1-R_{C}(i),\;$$

(3) $$S_{B}(i)=P+1-R_{B}(i),\;$$

(4) $$S_{i}=S_{D}(i)+S_{C}(i)+S_{B}(i),\;$$

Then, the LCI that captures the spatial changes of the port’s overall ranking in the GCSN is computed, taking into account the port’s latitude ($L_{i}$), as shown in Eq. (5).

(5) $$\mathrm{LCI}\;=\sum_{i\in P}S_{i}\times L_{i},\;$$

where P denotes the total number of ports.

It could be seen that the higher the value of LCI, the higher the center of the network is located, *i.e.* the whole network is shifting to the north. Regarding the analysis, the LCI of the GCSN without ASRs will be first calculated and used as a baseline for observing the changes in the network when the ASRs are introduced.

### Network assessment with ASRs

Three shipping routes were chosen for the assessment in this study, mainly because they traverse different continents in the Northern Hemisphere, offering strategic connections between major global markets. Specifically, Route A involves changing from the Panama Canal to NSR for the Yokohama-Halifax route, facilitating a direct connection between Asia and North America. Route B uses the NSR instead of the Suez Canal for the Rotterdam-Yokohama route, enhancing trade efficiency between Europe and Asia. Route C involves shifting from the Panama Canal to the NWP for the Hamburg-Seattle route, optimizing the link between Europe and North America. These routes were selected to illustrate the significant impact of Arctic shipping on intercontinental trade in the Northern Hemisphere (Theocharis et al., 2018). The ports of Yokohama, Halifax, Rotterdam, Hamburg, and Seattle are major hubs for international trade, making them critical points for assessing the benefits of these alternative Arctic routes. To properly assess the GCSN with ASRs, we have further modified the scenarios in this study to include the situations when each ASR is introduced,when all ASRs are fully commercialized, and some usages of original routes are switched to ASRs (Guo et al., 2022).

For Scenario 1, it is assumed that shipping routes are switching from the original routes to the new ASRs. It includes a reduction in the usage of original routes and an increment of TEUs to the new routes (*i.e.* the usage of original routes is incrementally reduced by 10%, and a multiple of 1,000 TEUs (1,000 TEUs, 2,000 TEUs, 3,000 TEUs, respectively) is added to the newroutes.. Three sub-scenarios associated with Route A, namely sub-scenarios 1A1 (Reduced by 10% and added 1,000 TEUs of the route usage), 1A2 (Reduced by 20% and added 2,000 TEUs of the route usage), and 1A3 (Reduced by 30% and added 3,000 TEUs of the route usage) will be explored. Likewise, sub-scenarios 1B1, 1B2, and 1B3, as well as 1C1, 1C2, and 1C3 are similarly constructed for Routes B and C.

For Scenario 2, it is assumed that capacity is only added to the new ASRs without reducing the usage of the original routes. It involves the situation in which capacity is added to the new routes without reducing the original route usage (Added 1,000 TEUs, 2,000 TEUs, and 3,000 TEUs, respectively), with three sub-scenarios, denoted by 2A1, 2A2, 2A3, 2B1, 2B2, 2B3, 2C1, 2C2, and 2C3.

Scenario 3 represents a scenario in which Arctic shipping is fully commercialized in some months, and this scenario assumes that cities of countries with territory in the Arctic Circle are added with new TEU capacities during the months when Arctic shipping is fully commercialized – with sub-scenarios 31, 32, and 33, denoting the situations at which 1,000 TEUs, 2,000 TEUs, and 3,000 TEUs are added to all new routes, respectively.

ASR scenarios, shown in Table 2 and explained in the previous paragraphs, are designed to investigate the potential effects of Arctic shipping and changes in capacity on liner shipping activities in the region, considering various route modifications and capacity adjustments applied during the summer season. Table 3 summarizes all changes and modifications according to all 21 ASR scenarios.

**Table 2.** A summary of all ASR scenarios.

Scenario	Sub-Scenario	Route	Original Route Reduction	TEUs Added to ASR
Scenario 1	1A1	Yokohama-Halifax	10%	1,000
Scenario 1	1A2	Yokohama-Halifax	20%	2,000
Scenario 1	1A3	Yokohama-Halifax	30%	3,000
Scenario 1	1B1	Rotterdam-Yokohama	10%	1,000
Scenario 1	1B2	Rotterdam-Yokohama	20%	2,000
Scenario 1	1B3	Rotterdam-Yokohama	30%	3,000
Scenario 1	1C1	Hamburg-Seattle	10%	1,000
Scenario 1	1C2	Hamburg-Seattle	20%	2,000
Scenario 1	1C3	Hamburg-Seattle	30%	3,000
Scenario 2	2A1	Yokohama-Halifax	0%	1,000
Scenario 2	2A2	Yokohama-Halifax	0%	2,000
Scenario 2	2A3	Yokohama-Halifax	0%	3,000
Scenario 2	2B1	Rotterdam-Yokohama	0%	1,000
Scenario 2	2B2	Rotterdam-Yokohama	0%	2,000
Scenario 2	2B3	Rotterdam-Yokohama	0%	3,000
Scenario 2	2C1	Hamburg-Seattle	0%	1,000
Scenario 2	2C2	Hamburg-Seattle	0%	2,000
Scenario 2	2C3	Hamburg-Seattle	0%	3,000
Scenario 3	3.1	All Routes	0%	1,000
Scenario 3	3.2	All Routes	0%	2,000
Scenario 3	3.3	All Routes	0%	3,000

**Table 3.** A summary of all ASR scenarios.

Route	Details of routes
A Sub-scenarios: 1A1, 1A2, 1A3, 2A1, 2A2, 2A3	Original route: Yokohama < -> Shanghai < -> Busan < -> Balboa < -> Cartagena < -> Kingston Jamaica < -> Miami < -> Savannah < -> New York/ New Jersey < -> Halifax New route: Yokohama < -> Shanghai < -> Busan < -> Vladivostok < -> Anchorage < -> Nuuk < -> Port of Argentia < -> Halifax
B Sub-scenarios: 1B1, 1B2, 1B3, 2B1, 2B2, 2B3	Original route: Rotterdam < -> Antwerp < -> Le Havre < -> Port Said < -> Jeddah < -> Port Klang < -> Singapore < -> Hong Kong < -> Shanghai < -> Yokohama New route: Rotterdam < -> Tromsø < -> Murmansk < -> Vladivostok < -> Busan < -> Shanghai < -> Yokohama
C Sub-scenarios: 1C1, 1C2, 1C3, 2C1, 2C2, 2C3	Original route: Hamburg < -> Le Havre < -> Southampton < -> New York/ New Jersey < -> Norfolk < -> Savannah < -> Miami < -> Balboa < -> Long Beach < -> Oakland < -> Seattle New route: Hamburg < -> Reykjavik < -> Port of Argentia < -> Nuuk < -> Anchorage < -> Vancouver < -> Seattle
Routes for Scenario 3 Sub-scenarios: 31, 32, 33	Bi-directional OD pairs are all set up between the following ports: Halifax, Port of Argentia, Vancouver, Shanghai, Hamburg, Aarhus, Nuuk, Reykjavik, Yokohama, Busan, Rotterdam, Oslo, Tromsø, Saint Petersburg, Murmansk, Vladivostok, Anchorage, New York/ New Jersey, and Seattle

## Results

Because of the fact that ASRs are sensitive to seasons and years, the analysis of GCSN with ASRs will be explored by both seasons and years. The LCIs and average degrees of such networks will be then reported, followed by the results of the GCSNs with ASRs emphasizing the changes caused by the ASRs.

### Ranking of ports in the GCSN without ASRs by seasons

Table 4 reports the top 20 ports in the Global Container Shipping Network (GCSN) without Arctic Shipping Routes (ASRs), as measured by their overall rank across different seasons. The ranking of these ports is based on their latitude and prominence in the shipping network during winter, spring, summer, and autumn. The ports are listed according to their rank for each season, reflecting their importance and activity level throughout the year. This detailed ranking by season highlights the dynamic nature of port activities and their varying significance throughout the year. It provides a comprehensive view of the top ports in the GCSN without ASRs, showcasing the importance of these ports in global trade networks regardless of seasonal changes.

**Table 4.** Top 20 ports as measured by the overall rank by seasons.

Rank	Winter		Spring		Summer		Autumn	
	Port	Latitude (° N)	Port	Latitude (° N)	Port	Latitude (° N)	Port	Latitude (° N)
1	Singapore	1.2833	Singapore	1.2833	Singapore	1.2833	Singapore	1.2833
2	Rotterdam	51.9	Rotterdam	51.9	Port Klang	3	Rotterdam	51.9
3	Port Klang	3	Port Klang	3	Rotterdam	51.9	Port Klang	3
4	Hong Kong	22.2667	Shanghai	31.2167	Shanghai	31.2167	Shanghai	31.2167
5	Busan	35.1	Hong Kong	22.2667	Busan	35.1	Busan	35.1
6	Algeciras	36.1333	Busan	35.1	Hong Kong	22.2667	Hong Kong	22.2667
7	Tanjung Pelepas	1.3619	Tanjung Pelepas	1.3619	Algeciras	36.1333	Algeciras	36.1333
8	Antwerp	51.2333	Algeciras	36.1333	Tanjung Pelepas	1.3619	Tanjung Pelepas	1.3619
9	Shanghai	31.2167	Antwerp	51.2333	Antwerp	51.2333	Antwerp	51.2333
10	New York NY/NJ	40.6941	Le Havre	49.4833	Le Havre	49.4833	Le Havre	49.4833
11	Le Havre	49.4833	Yantian	22.5833	Valencia	39.45	Yantian	22.5833
12	Yantian	22.5833	Kaohsiung	22.5653	Ningbo	29.8667	Kaohsiung	22.5653
13	Kaohsiung	22.5652	New York NY/NJ	40.6942	New York NY/NJ	40.6942	New York NY/NJ	40.6942
14	Hamburg	53.55	Valencia	39.45	Kaohsiung	22.5653	Ningbo	29.8667
15	Bremerhaven	53.55	Hamburg	53.55	Yantian	22.5833	Valencia	39.45
16	Valencia	39.45	Bremerhaven	53.55	Tanger	35.7833	Hamburg	53.55
17	Manzanillo	9.3605	Tanger	35.7833	Hamburg	53.55	Tanger	35.7833
18	Dubai, Jebel Ali	24.9958	Manzanillo	9.3605	Bremerhaven	53.55	Dubai, Jebel Ali	24.9958
19	Tanger	35.7833	Colombo	6.95	Colombo	6.95	Manzanillo	9.36053
20	Ningbo	29.8667	Jeddah	21.4586	Manzanillo	1.2833	Bremerhaven	53.55

From Table 4, Singapore consistently holds the top position throughout all seasons, while Rotterdam remains the second most central port in almost all seasons except the summer. Port Klang maintains the third central port during winter and spring but drops to the fourth in summer and autumn. Hong Kong secures the fourth rank during summer and autumn but considerably slips to the sixth in winter and spring, presumably because of the intense freight flow from Asia to Europe before the fall festive seasons. Busan consistently holds the fifth position. Algeciras holds the sixth position during summer and winter, slightly falling to the seventh in winter and spring. Likewise, Tanjung Pelepas remains the seventh most central port in winter, spring, and summer, but it slightly drops to the eighth place during winter. Antwerp consistently ranks as the eighth most central port, while Shanghai maintains the ninth position during summer and winter, rising to the fourth place in winter and spring. New York holds the tenth position during winter, spring, and summer, but it largely drops to the thirteenth in winter. Other ports also experience variations in rankings across different seasons with constant latitudes.

During the summer, there is a noticeable trend of some ports shifting towards higher latitudes due to more favorable sailing conditions in the north. While Singapore maintains its top position with a latitude of 1.2833° N, ports, such as Shanghai, experience a significant rise in the ranking, moving from the fourth to the second place with the same latitude of 31.2167° N. On the contrary, Hong Kong drops to the sixth position with a latitude of 22.2667° N during the summer. These shifts suggest a potential seasonal pattern in their rankings, with some ports performing relatively better or worse during summer. Not all ports exhibit this northward shift, as various factors – including trade patterns, weather conditions, and shipping preferences during the summer – induce these seasonal variations.

In addition to the overall rank, Table 5 provides information on the LCI and the average degree of GCSN without ASRs by season. From Table 5, both LCI and average degree values are higher in the summer and autumn compared to the remaining seasons. This suggests that during the summer and autumn seasons, the GCSN is more connected, and its central location seems to shift to the northern position.

**Table 5.** LCI and average degree by seasons.

Index	Winter	Spring	Summer	Autumn
LCI	10283.4943	10291.8505	10374.7686	10357.3411
Average degree	5.671	5.761	5.812	5.811

### Ranking of ports in the GCSN without ASRs by years

Table 6 reports the top 20 ports in the GCSN without ASRs, as measured by the overall rank by years.

**Table 6.** Top 20 ports as measured by the overall rank by years.

Rank	2012		2013		2014		2015		2016	
	Port	Latitude (° N)	Port	Latitude (° N)	Port	Latitude (° N)	Port	Latitude (° N)	Port	Latitude (° N)
1	Singapore	1.2833	Singapore	1.2833	Singapore	1.2833	Singapore	1.2833	Singapore	1.2833
2	Port Klang	3	Hong Kong	22.2667	Shanghai	31.2167	Rotterdam	51.9	Port Klang	3
3	Hong Kong	22.2667	Busan	35.1	Rotterdam	51.9	Port Klang	3	Busan	35.1
4	Busan	35.1	Port Klang	3	Busan	35.1	Shanghai	31.2167	Rotterdam	51.9
5	Shanghai	31.2167	Shanghai	31.2167	Port Klang	3	Busan	35.1	Shanghai	31.2167
6	Tanjung Pelepas	1.3619	Tanjung Pelepas	1.3619	Tanjung Pelepas	1.36194	Tanjung Pelepas	1.3619	Algeciras	36.1333
7	Rotterdam	51.9	Rotterdam	51.9	Algeciras	36.1333	Algeciras	36.1333	Tanjung Pelepas	1.3619
8	Algeciras	36.1333	Algeciras	36.1333	Hong Kong	22.2667	Antwerp	51.2333	Antwerp	51.2333
9	Kaohsiung	22.5653	Kaohsiung	22.5653	Antwerp	51.2333	Hong Kong	22.2667	Le Havre	49.4833
10	Antwerp	51.2333	Le Havre	49.4833	Le Havre	49.4833	Le Havre	49.4833	Hong Kong	22.2667
11	Yantian	22.5833	New York NY/NJ	40.6942	Kaohsiung	22.5653	Ningbo	29.8667	Kaohsiung	22.5653
12	Le Havre	49.4833	Antwerp	51.2333	New York NY/NJ	40.6942	Kaohsiung	22.5653	Ningbo	29.8667
13	New York NY/NJ	40.6942	Manzanillo	9.3605	Bremer-haven	53.55	New York NY/NJ	40.6942	Colombo	6.95
14	Valencia	39.45	Savannah GA	32.0833	Hamburg	53.55	Hamburg	53.55	Tanger	35.7833
15	Manzanillo	9.36054	Jeddah	21.4586	Port Said	22.5833	Tanger	35.7833	New York NY/NJ	40.6942
16	Santos	−23.95	Yantian	22.5833	Tanger	35.7833	Xiamen	24.45	Hamburg	53.55
17	Balboa	8.9576	Ningbo	29.8667	Jeddah	53.55	Yantian	22.5833	Valencia	39.45
18	Ningbo	29.8667	Tanger	35.7833	Manzanillo	53.55	Valencia	39.45	Dubai, Jebel Ali	24.9958
19	Tanger	35.7833	Port Said	31.2533	Savannah GA	6.95	Bremerhaven	53.55	Bremerhaven	53.55
20	Jeddah	21.4586	Dubai, Jebel Ali	24.9958	Valencia	1.2833	Savannah GA	32.0833	Qingdao	36.0959

From Table 6, it can be observed that specific ports on the list exhibit a noticeable shift towards higher latitudes, indicating a northward movement. Furthermore, Singapore consistently maintains its top position throughout the years, with a latitude of 1.2833° N. Port Klang also remains in second place across multiple years, with a latitude of 3° N. Hong Kong shows some variation in its ranking, but it generally maintains a relatively stable position at a latitude of 22.2667° N. Likewise, Busan is relatively stable regarding both ranking and latitude.

Unlike the abovementioned ports, Shanghai demonstrates a significant shift in both its ranking and latitude, moving from the fifth most-central position in 2012 to the second most-central position in 2015 and 2016, with a latitude of 31.2167° N. Algeciras maintains a relatively stable rank and remains situated at around 36.1333° N. Rotterdam consistently holds a high rank and remains at a latitude of 51.9° N. Kaohsiung experiences some variations in ranking. However, it usually remains at a latitude of approximately 22.5653° N. Similarly, Antwerp maintains a relatively stable rank and latitude at 51.2333° N. Ports such as Le Havre and New York NY/NJ exhibit similar ranks in multiple years, with their latitudes remaining relatively consistent.

In addition to the overall rank, Table 7 provides information on the LCI and the average degree of GCSN without ASRs by years.

**Table 7.** LCI and average degree by years.

Index	2012	2013	2014	2015	2016
LCI	8727.2551	9068.7819	9729.3180	9741.2716	9754.6554
Average degree	3.126	3.305	3.676	3.938	4.018

Table 7 shows a rise in the LCI values from 2012 to 2016, indicating a gradual growth in such an index. The average degree also shows an increasing trend over the same period, suggesting that the GCSN has experienced an overall increase in connectivity and complexity over the years.

It is worth remarking that the results from Sections 4.1 and 4.2 provide valuable insights into the variation of the shipping networks over different seasons and years. By analysing ports’ rankings and centrality measures, we can observe a growing interest in opening new shipping routes in the north. Seasonal and annual variations highlight how certain ports gain prominence during specific times, suggesting favorable conditions for northern routes. This trend underlines the potential and emerging significance of ASRs in enhancing global trade connectivity, reflecting an adaptive and evolving maritime network that increasingly considers northern passages.

### Impact of ASRs on the GCSN

Table 8 presents the LCI values and the average degrees of GCSN according to all 21 ASR scenarios. Across sub-scenarios 1A, 1B, 1C, 2A, 2B, and 2C, the LCI values exhibit minor variations, suggesting that these configurations do not significantly alter the network’s centrality, with values ranging narrowly between 10359.3945 and 10370.0391. In contrast, Scenario 3 (sub-scenarios 31, 32, 33) shows a noticeable decrease in LCI values to a range of 10229.2159–10241.9122, indicating a substantial impact on the network’s centrality due to the inclusion of Arctic shipping routes. Additionally, while the average degree remains consistent at 5.818 across most sub-scenarios, it increases to 6.116 in Scenario 3, reflecting enhanced connectivity and resilience within the network.

**Table 8.** LCI and average degree with ASR routes.

Sub-scenarios	1A1	1A2	1A3	Average degree
LCI	10369.7587	10366.4203	10364.0072	5.818
Sub-scenarios	1B1	1B2	1B3	
LCI	10366.5820	10361.5002	10359.4841	5.818
Sub-scenarios	1C1	1C2	1C3	
LCI	10369.1892	10365.3526	10363.1003	5.820
Sub-scenarios	2A1	2A2	2A3	
LCI	10369.7076	10366.3299	10364.0072	5.818
Sub-scenarios	2B1	2B2	2B3	
LCI	10366.5478	10361.4659	10359.3945	5.818
Sub-scenarios	2C1	2C2	2C3	
LCI	10370.0391	10367.1658	10365.3653	5.822
Sub-scenarios	31	32	33	
LCI	10241.9122	10233.6825	10229.2159	6.116

For sub-scenarios 1A, 1B, 1C, 2A, 2B, and 2C, the changes in LCI values are minimal, indicating that small-scale adjustments or incremental changes to the network do not significantly affect its centrality. The results suggest consistency in the network’s structure, maintaining its centrality and overall connectivity. Additionally, the stability of network resilience is evident as the average degree remains constant at 5.818, indicating that the network’s ability to handle disruptions or reroute traffic efficiently does not show significant improvement under these sub-scenarios.

In contrast, Scenario 3 (sub-scenarios 31, 32, 33) shows a more pronounced impact on the network. The significant decrease in LCI values (down to 10229.2159) suggests a shift in the network’s centrality, likely due to the increased importance of new routes introduced by Arctic shipping. Furthermore, the increase in the average degree to 6.116 reflects enhanced connectivity, as more routes are being utilized, leading to a denser and potentially more resilient network. This enhanced network resilience means the network can better withstand disruptions as more alternative routes are available for rerouting traffic.

The analysis reveals that minor network adjustments do not significantly impact the centrality or resilience of the global shipping network. However, the incorporation of Arctic shipping routes, as represented in Scenario 3, substantially shifts the network’s centrality and increases the average degree, thereby enhancing the network’s resilience and connectivity. The finding underscores the necessity for fundamental network changes to achieve a more resilient and better-connected global shipping network.

Considering the augmented interconnectivity by accounting for ASRs would be natural. Critical thinking about the explicit pathways through which ASRs influence the GCSN is encouraged. Additionally, assessing the performance of LCI compared to other metrics or exploring ways to validate the proposed LCI would be beneficial. It involves analysing how ASRs might change the dynamics of the network, potentially leading to new patterns of connectivity and centrality. A more detailed examination of these aspects could provide deeper insights into the implications of integrating ASRs into global shipping networks.

## Discussion and implications

Container shipping operators can derive valuable insights from the outcomes of this research, as it enhances their comprehension of the possible alterations in global shipping networks resulting from the integration of ASRs into the industry. Furthermore, the assessment framework established in this paper empowers these operators to gauge the centrality of various ports within the network and concurrently evaluate the impact of ASRs on their levels of connectivity and accessibility. With this information, shipping lines can make informed decisions regarding route optimization, resource allocation, and strategic planning. They can identify new opportunities or challenges from opening ASRs and adapt their operations accordingly to ensure operational sustainability.

Besides container shipping operators, port operators stand to gain valuable insights from this research. As this study examines the resilience of the GCSN – and that of a port – as a result of ASRs at both seasonal and annual levels, port operators can better assess the potential changes in traffic flow, cargo volumes, and connectivity patterns. This information can help guide port investment decisions like infrastructure development, capacity expansion, and service offerings. Moreover, port operators can strategically position themselves to capitalize on the changing dynamics of the shipping network and attract more container vessels utilizing ASRs.

In conclusion, this study furnishes scientific evidence of climate change’s influence on shipping networks and provides valuable perspectives on the prospective consequences of ASRs within the GCSN. Introducing this assessment framework equips stakeholders with the means to boost their operational efficiency, optimize resource allocation, and secure the sustainability of their endeavors amidst climate-induced alterations in shipping patterns.

## Conclusion

In conclusion, the analysis of GCSN with the commercialization of ASRs has provided valuable insights into the dynamics and resilience of the network. While specific ports have shown a shift towards higher latitudes, indicating potential seasonal variations and factors specific to each port’s performance, it is evident that not all ports exhibit this trend. This emphasizes the need to consider various factors, such as trade patterns and global dynamics, when assessing the performance and importance of ports.

This research adds significant value to the field by introducing a comprehensive centrality assessment for global shipping networks, encompassing Arctic shipping. The outcomes underscore the importance of Arctic shipping in fortifying the network’s resilience and mitigating climate-related vulnerabilities stemming from climate change. These results can serve as a fundamental framework for liner shipping companies to formulate novel shipping routes and fine-tune their cargo capacities, leveraging the prototype model developed in this study.

To further advance the research in this area, future studies could explore the integration of local climate vulnerability indicators and other indicators related to port resilience, including economic and political aspects. Further studies would provide a more comprehensive understanding of the impact of Arctic shipping on the entire shipping network and enable decision-makers to make informed choices regarding investments and critical infrastructure development. Additionally, future research could incorporate more extensive databases and employ advanced big data techniques to conduct more detailed analyses and gain deeper insights into the complex dynamics of global shipping networks.

Promoting and investing in the development of Arctic shipping and related technologies are feasible and crucial for addressing the challenges posed by climate change and enhancing the overall resilience of the global shipping network. Furthermore, conducting a weight network analysis with cost comparison would further contribute to understanding the influence of Arctic shipping on the entire shipping network, facilitating more informed decision-making in the industry. By continuously exploring these research directions and addressing the complexities of global shipping networks, we can enhance our understanding of the interplay between Arctic shipping, climate vulnerabilities, and network resilience, ultimately fostering sustainable and efficient maritime transportation in a changing global environment.

## Supplementary Material

Supplemental Material

Supplemental Material
