# Supplementary material for: Assessing the impact of Arctic shipping routes on the global container shipping network’s connectivity
Source: Polar Geogr (Palm Beach). 2024 Sep 11;47(3):219–39. doi: 10.1080/1088937X.2024.2399775 (PMC11441395; doi:10.1080/1088937X.2024.2399775)
Supplement: Supplemental Material [file TPOG_A_2399775_SM7561.docx]

Appendix 2

The algorithm for calculating three centrality measures is presented first to understand the vulnerability assessment mechanism. Based on the graph theory, a network is built by ports and links. The structure of a network with $N$ ports can be presented by a $N\times N$ binary matrix. $\left( W \right):$

$W=\left[ \begin{matrix} w_{11} & \cdots& w_{1N} \\ \vdots& \ddots& \vdots\\ w_{N1} & \cdots& w_{NN} \end{matrix} \right]$ (1)

The unnormalised weight$w_{ij}$ is 0, assuming that there is no connection between ports$i$ and$j$. In the case of undirected networks with no loops, the adjacency matrix is symmetric$(w_{ij}=w_{ji}\geq0)$, and all elements of the main diagonal, from $i$ to $i$, equal 0 ($w_{ii}=0$).

Degree centrality is the first invented and calculated by the number of links connecting to a port, as shown in Equation (2) and Equation (3). In-degree ($D(in)$) is the number of connections that point inward at a vertex, and out-degree ($D(out)$) is the number of connections that originate at a vertex and point outward to other vertices. The lesser the degree value of a port, the less it is connected to or from other ports, and vice versa.

${D(in)}_{i}=\sum_{h}^{N} w_{hi}$ (2)

${D(out)}_{i}=\sum_{j}^{N} w_{ij}$ (3)

$N$ represents the total ports within an investigated network, $w_{hi}$ represents the weighted link from $h$ to $i$, and $w_{ij}$ represents the weighted link from $i$ and $j$. In-closeness ($C(in)$) and out-closeness ($C(out)$) of a port are computed by Equation (4) and Equation (5) by dividing the number of the port in the network except for $i$ ($N-1$) by the length of the shortest path length between $i$ and other ports in the network.

${C(in)}_{i}=\frac{N-1}{{D(in)}_{i}}=\frac{N-1}{\sum_{h}^{N} w_{hi}}$ (4)

${C(out)}_{i}=\frac{N-1}{{D(out)}_{i}}=\frac{N-1}{\sum_{j}^{N} w_{ij}}$ (5)

Betweenness centrality (B) considers a port amid other port pairs illustrated in the diagram, showing the role of the port in the network. Since intermediate points control the linkage between these ports, the higher the betweenness ports, the more influential ports are. It can be defined by adopting Equation (6) where $s, t$ represents a port pair, $w(s,t|i)$ is the number of times that $s$ and $t$ cross the port $i$ with the shortest interval and $w(s,t)$ is the total number of shortest paths between $s$ and $t$.

$B_{i}=\sum_{\begin{aligned} s,t\in N \\ s,t\neq i \end{aligned}} \frac{w\left( s,t|i \right)}{w\left( s,t \right)}$ (6)

Three centrality measures are integrated by employing a Borda Count approach. The higher values calculated by Equations (2) – (6), the higher ranks of cruise ports are obtained. $R_{D\left( in \right)}, R_{D\left( out \right)}, R_{C\left( in \right)},R_{C\left( out \right)},R_{B}$ refer to the ranking position of all the cruise ports in the ASEAN countries by Equation (2) – (6), where the ranks for$D\left( in \right),D\left( out \right), C\left( in \right), C\left( out \right),B$ can be obtained independently. The count score ($S_{D\left( in \right)}\left( i \right), S_{D\left( out \right)}\left( i \right), S_{C\left( in \right)}\left( i \right),S_{C\left( out \right)}\left( i \right),S_{B}\left( i \right)$) is provided by the ranking order of every centrality for the *i^th^* investigated ports as shown in Equations (6) to (10).

$S_{D\left( in \right)}\left( i \right)=N-R_{D\left( in \right)}\left( i \right)+1$ (7)

$S_{D\left( out \right)}\left( i \right)=N-R_{D\left( out \right)}\left( i \right)+1$ (8)

$S_{C\left( in \right)}\left( i \right)=N-R_{C\left( in \right)}\left( i \right)+1$ (9)

$S_{C\left( out \right)}\left( i \right)=N-R_{C\left( out \right)}\left( i \right)+1$ (10)

$S_{B}\left( i \right)=N-R_{B}\left( i \right)+1$ (11)

The ranks of degree and closeness centrality measures involve two directions together. A one-third count score of each centrality is used for the final result to ensure a fair contribution by degree, closeness, and betweenness centrality. Accordingly, $S_{D\left( in \right)}\left( i \right), S_{D\left( out \right)}\left( i \right), S_{C\left( in \right)}\left( i \right) and S_{C\left( out \right)}\left( i \right)$ indicate one-sixth to the final score independently, as they are bilateral pairs. Then, $S_{B}\left( i \right)$ indicates one-third of the result as it is not bilateral. The related fair contribution of a directional indicator is shown in Equation (12) and Equation (13):

$S_{D}\left( i \right)=\frac{S_{D(in)}\left( i \right)+S_{D(out)}\left( i \right)}{2}$ (12)

$S_{C}\left( i \right)=\frac{S_{C(in)}\left( i \right)+S_{C(out)}\left( i \right)}{2}$ (13)

By obtaining the overall rank, the significance of a port to the global shipping network can be presented, and the overall rank score ($S_{O}$) may be obtained by Equation (14):

$S_{O}\left( i \right)=S_{D}\left( i \right)+S_{C}\left( i \right)+S_{B}\left( i \right)$ (14)
